# Differences in stress defence mechanisms in germinating seeds of *Pinus sylvestris* exposed to various lead chemical forms

**DOI:** 10.1371/journal.pone.0238448

**Published:** 2020-09-28

**Authors:** Aleksandra Maria Staszak, Arleta Małecka, Iwona Ciereszko, Ewelina Ratajczak

**Affiliations:** 1 Laboratory of Plant Physiology, Department of Plant Biology and Ecology, Faculty of Biology, University of Bialystok, Bialystok, Poland; 2 Laboratory of Biotechnology, Institute of Molecular Biology and Biotechnology, Adam Mickiewicz University, Poznań, Poland; 3 Institute of Dendrology, Polish Academy of Sciences, Kórnik, Poland; Government College University Faisalabad, PAKISTAN

## Abstract

Exposure to lead (Pb) can have serious toxic effects on the physiological and biochemical processes of plants. The chemical form of the metal determines the degree of its toxicity. In our research, we examined the effect of lead in the form of lead nitrate [Pb(NO_3_)_2_] and lead chloride (PbCl_2_) in concentrations of 12.5 mM and 25 mM on pine (*Pinus sylvestris*) seed germination. Nitrogen salt causes more severe changes than chloride salt. Increasing levels of electrolyte leakage, malondialdehyde, and hydrogen peroxide were detected during germination processes. The high levels of ROS lead to redox changes in the cell. We observed a reduction in the level of the reduced form of glutathione (GSH), and at the same time observed increased levels of the oxidised form of glutathione (GSSG) depending on the concentration and also the time of exposure to lead compounds. At the beginning of germination processes, the effective non-enzymatic activity of the antioxidant cycle was dominant, and at the late stage the enzymatic activity was noticed in the presence of Pb compounds. CAT activity significantly increased after Pb compound exposition.

## Introduction

Germination is an important and complex process that ends with seedling emergence [1]. Seeds produced by plants from temperate climate zones, before natural shedding, have developed tolerance to desiccation. The anabiosis phase ends in non-dormant seeds when proper environmental and physiological conditions for germination occur. Germination starts with imbibition; then the catabolic and anabolic phase is required for seedling formation [1]. The process of germination could be inhibited by a natural occurrence, such as dormancy, but also by abiotic factors like drought or pollution. Heavy metal concentrations in the environment including lead (Pb), increase year by year and have a negative impact not only on human health, but also on the proper functioning of ecosystems [2]. Pb is currently considered as one of the most harmful substances and contamination with it is very dangerous due to its non-biodegradability and widespread use [3]. Pb is toxic to plants at even low concentrations [4] and is easily absorbed and accumulated in various parts of the plant. A high concentration can cause several toxic symptoms in plants including growth retardation, a negative effect on leaf size and colour (chlorosis), inhibition of photosynthesis, disruption of mineral nutrition and water balance, and changes in hormonal status as well as membrane structure and permeability [5]. This article considers the effect of Pb ions in two different compounds [lead nitrate—PbCl_2_ and lead chloride Pb(NO_3_)_2_] on seed germination, seedling growth and metabolism. The bioavailability of lead is influenced by many factors such as chemical compound type, soil type and soil pH. The lead compounds used here–Pb(NO_3_)_2_ and PbCl_2_ –are among the chemically active fractions that are easily absorbed by plants, unlike, e.g, lead phosphate [Pb_3_(PO_4_)_2_], lead sulphate (PbSO_4_), lead sulphide (PbS), and lead chloride phosphate [Pb_5_(PO_4_)_3_Cl], which make the lead inaccessible and therefore pose less of a potential risk to the biota [6]. Different concentrations of Pb compounds were used to look for changes at various levels–not only macroscopic, but also cellular which was toxic but not lethal to plants. It has been proven so far that heavy metals, including Pb, in germinating seeds cause: reducing their nutrient reserves by immobilizing starch as a result of reducing or inhibiting proteases and α-amylases [7], inhibiting germination by hindering water absorption [8], delaying seedling growth, decreasing germination percentage, germination index, root /shoot length, tolerance index and dry mass of roots and shoots, generating oxidative stress and decreasing chlorophyll content [9]. To date, the toxicity of Pb and various other ions such as Cu, Zn, Cd and Hg, has been studied in accordance with seed germination and the seedling emergency test, mainly for monocotyledons [8] or the Arabidopsis model plant [10], though relatively few tests have been conducted for different trees [11–14]. However, the above-mentioned studies focus mainly on the growth parameters of seedlings, and not on biochemical and physiological changes in germinating seeds. The literature doe not contain much data on the effect of heavy metals (Pb, Cd, Cu) on germinating seeds hence, the need to investigate this topic.

Pb cause oxidative stress, which influences different metabolic pathways and growth processes in the plant. The redox state shift in cells induces the generation of reactive oxygen species (ROS): hydrogen peroxide (H_2_O_2_) and superoxide anion (O_2_^.-^). The regulation of ROS levels is usually controlled via the efficient activity of antioxidant systems [15]. The antioxidant system consists of non-enzymatic and enzymatic pathways. The non-enzymatic components include glutathione (GSH) and ascorbic acid, non-protein amino acids, phenolic compounds, α-tocopherols, etc. The main enzymatic components include antioxidant enzymes such as: catalase (CAT, EC 1.11.1.6), ascorbate peroxidase (APX, EC 1.11.1.11), and superoxide dismutase (SOD, 1.15.1.1), which are involved in dismutation or ROS detoxification. Catalase plays a crucial role in the removal of hydrogen peroxide and protects the cell from oxidative stress. Its special properties include the fact that has one of the highest turnover numbers as one molecule of enzyme hydrolyses more than one million molecules of the substrate per second and has a fairly broad range of working from 4 to 11 optimum pH [16]. The control of seed germination by CAT is confirmed in dormant seeds wherein an *Arabidopsis thaliana* mutant defective in CAT activity germinates promptly in suitable conditions. A decrease in CAT activity and the consequent rise in the H_2_O_2_ level were related to germination signalling [17]. Other authors [18] showed in germinating seeds, metabolic activity with continuously high ROS production have CAT activity that controls the process of seed transition from dormancy to germination. Thiol metabolism plays a crucial role in heavy metal detoxification though the formation of mercaptide bonds with metal groups because of their nucleophilic nature. The sequestration of toxic metals–for example, Pb *via* thiol groups of GSH–protects plant cells. The cellular ROS level may be regulated during stress by GSH–playing a major biochemical role as a water-soluble non-protein thiol component [19, 20]. The diverse role of GSH is present in different developmental processes, and gene expression under the realisation of the growth program, but also under abiotic stress conditions. Nowadays, glutathione half-cell redox potential (E_GSSG/2GSH_) is considered to be a marker of seed viability [21, 22]. The exact mechanism of GSH in stress conditions, including heavy metal influence, still requires more studies, especially because oxidative stress is the most severe for proper plant metabolism [23].

Scots pine (*Pinus sylvestris* L.) is one of the most common tree species planted across Europe in the forest industry. Pine seeds are used in forest nurseries for seedling establishment and reforestation, for example in a polluted area [24]. It is very important to understand how Pb influences the regulation of the redox state during germination processes and seedling formation in plants with a long life cycle. Not much is known about the biochemical and physiological processes occurring in germinated seeds under the influence of Pb even though the growth parameters of different plant species have been studied. The main aim of our research was to determine: i) how various Pb compounds influence different stages of Scots pine seed germination, ii) which Pb compound and in what concentration is more phytotoxic for germinating seeds, and iii) which line of defence and antioxidant system components are the most important in the subsequent germination stages under the toxic effects of Pb.

## Materials and methods

### Plant material

Scots pine (*Pinus sylvestris* L.) cones were collected in January of 2018 from the Czarna Białostocka District of the Polish Forest (north-eastern Poland). The experiments were conducted in 2019 under laboratory conditions. The germination test was conducted in four replications (50 seeds on each Petri dish). During the experiment, we used lead chloride (PbCl_2_) and lead nitrate [Pb(NO_3_)_2_], with both compounds in concentrations of 12.5 mM and 25 mM. The reported concentrations of Pb compounds were selected several other concentrations affecting seed germination–in preliminary experiments. For research design concentrations that reduced germination in 50% were taken. After 1, 3 or 7 days of treatment, the seeds were collected and frozen for further analysis (-80°C).

### Germination studies

*Energy of germination* (EG) was calculated on the seventh day of germination using 50 seeds in four replicates, witch each experiment involving control (distilled water), and research treatment of both Pb compounds in two concentrations (12.5mM and 25 mM). The germination test was carried out in Petri dishes in a growth chamber with an 8/16 day/night period, with the daytime temperature reaching 24 ᵒC, falling to 20 ᵒC at night, with 70% humidity. Photosynthetic active radiation (PAR) was 120 μM m^-2^ s^-1^. We regarded seeds as germinated when a root length of 3 mm had been achieved. In the control plants we observed germination of nearly 80%. The results were shown as percentage of germination.

### Electrolytes leakage (EL)

For each measurement, 10 seeds were used. During the first stage, we put seeds from each Pb treatment in deionised water (twice for 15 min) to extract heavy metal ions from the seed coat and to remove other surface-adhered electrolytes. Then, each sample was put into 25 ml of deionised water and incubated at room temperature for 1h initial measurements of conductivity (E) were noted. All measurements were made with a conductometer GRYF 158 (GRYF HB, Ltd., Czech Republic). At the next stage, the seeds were frozen at -20°C and then incubated for 1h in room temperature to measure total electrolyte leakage (EF). Electrical conductivity (EL) was calculated from EL = (E/EF) × 100 (%). Each measurement was taken three times per sample.

### Root length measurements

To calculate how Pb compounds influence seedling emergence, we measured root length. During the germination test, we took a photo of each variant and then analysed it via the ImageJ program. The results were shown according to 10 measurements for each variant after 1, 3 or 7 days of germination.

### Oxidative stress parameters

#### Superoxide anion (O_2_^•−^) determination

The O_2_^•−^ level was measured according to the reduction of nitro blue tetrazolium (NBT) with the [25] method. Samples were incubated with 3 mL of 0.05 M K-phosphate buffer (pH 7.8) with 0.05% NBT and 10 mM sodium azide, at room temperature without light, for 30 min. Then, each variant was heated in a water bath to 85°C for 15 min, and then put on ice. The absorbance was read at 530 nm (Spectrophotometer DU 730, Beckman Coulter Life Sciences, California, USA). The O_2_^•−^ formation was shown as ΔA_530_ g^−1^ DW of the sample. *Localisation of superoxide* was carried out according to [26]. Seeds from each treatment (10 per sample) were put into a 0.2% solution of nitro blue tetrazolium chloride (NBT, Sigma Chemical Co) in a 50 mM sodium phosphate buffer (pH 7.5) and incubated at room temperature for 24 h (supplementary material).

#### Hydrogen peroxide (H_2_O_2_) content

The H_2_O_2_ level was determined according to the ferrithiocyanate method of [27]. For analysis, 0.2 g of seeds during germination were homogenised in 5 mL of 5% (w/v) trichloroacetic acid (TCA) with 10 mM EDTA. The final volume of H_2_O_2_ content was shown as nM x g^-1^ FW. *Localisation of hydrogen peroxide* was carried out according to [28]. Seeds from each treatment (10 per sample) were immersed in a 1% 3,3’-diaminobenzidine solution (DAB, Sigma Chemical Co.) in water for 24h at room temperature. (supplementary material).

#### Evaluation of malondialdehyde (MDA)

Samples of 0.2 g of seeds per variant were homogenised in 5 ml of 5% (w/v) trichloroacetic acid (TCA, POCH Chemical Co.) solution containing 10 mM/L EDTA (Sigma Chemical Co.). MDA content was measured according to [29]. The supernatant was mixed with thiobarbituric acid (TBA, Sigma Chemical Co.) to determine the malondialdehyde content. Levels of MDA were calculated as the difference of absorbance A_532_–A_600_ and shown as nM x g^-1^FW.

### Enzymatic antioxidant acitivity

#### Catalase (CAT) activity

CAT was extracted according to [30]. The samples were homogenised in a cooled mortar with an extracting buffer (1.25 mM PEG 4000, 0.1 mM EDTA, 2mM DTT and 0.1 M phosphate buffer pH 728) with 0.2 g of PVP. The kinetic reaction was analysed by directly measuring the decomposition of H_2_O_2_ at 240 nm (Beckman Coulter DU 730, England). The results were expressed as specific activity, i.e. as nM H_2_O_2_ decomposed min^-1^ mg^-1^ protein corresponding to the means ± SD of the values obtained with measurements carried out on three different extracts.

#### Protein content

Seeds from the germinating tests (0.2 g) were homogenised in a buffer of 200 mM Tris–HCl, pH 7.5; 5% glycerol and 1 mM phenylmethyl-sulfonyl fluoride (PMSF, Sigma Chemical Co.). The protein concentration was estimated according to [31] with BSA as the standard.

### Redox state

#### Glutathione assays

Glutathione in two forms–the reduced (GSH) and the oxidised (GSSG)–was determined according to [32]. Samples of 0.2 g of *P*. *sylvestris* seeds were homogenised in an ice cooled mortar with 5% (w/v) sulfosalicylic acid (Sigma Chemical Co.), after which the samples were centrifuged at 10 000g for 20 min. A 1 mL aliquot of the supernatant was removed and neutralised by adding 1.5 mL of 0.5 M/L K-phosphate buffer (pH 7.5). This sample was used to determine the amount of total glutathione (GSH+GSSG). Another 1 mL of neutralised supernatant was pre-treated with 0.2 mL of 2-vinylpyridine for 1.5 h at 25°C to mask GSH and to enable GSSG to be determined alone. Both samples were extracted twice with 5 mL of diethyl ether. The incubation mixture contained: 0.5 mL of 0.1 mM/L Na-phosphate buffer (pH 7.5) containing 5 mM/L EDTA, 0.2 mL of 6 mM/L 5.5′-dithiobis-(2-nitrobenzoic acid, Sigma Chemical Co.), 0.1 mL of 2 mM/L NADPH, 0.1 mL (1 unit) of GR type III (Sigma Chemical Co.), and 0.1 mL of extract. The change at 412 nm was followed at 25°C. A standard curve was prepared through utilisation of the GSH standard.

#### Glutathione half cell reduction potential (E_GSSG/2GSH_)

Reduced (GSH) and oxidised (GSSG) forms of glutathione were determined using the procedure by [32] described above. From GSH and GSSG levels, E_GSSG/2GSH_ was calculated as described by [21] using the Nernst equation E_GSSG/2GSH_  =  E0 − RT/nFln [GSH]2/[GSSG], where *R* is the gas constant, *T* is the temperature in *K*, *F* is the Faraday constant, *n* is the number of transferred electrons, and E0 is the standard half-cell reduction potential of glutathione (− 0.240 mV) at pH 7.5.

### Statistical analysis

For the statistical analysis of the biochemical results, we used ANOVA and the Duncan post-hoc test, p <0.05. The mean values ± S.E were shown in each figure. The data were analysed via Statistica (StatSoft Poland).

## Results

### Germination test, root length

In the experiment the first, third and seventh days of germination were considered. On the seventh day of control, various well developed seedlings were observed in the control treatment. The energy of germination varied depending on the concentration used; in the control seeds, germination was observed on the third day, in the concentration of 12.5 mM on the fourth day, and on the fifth day for the 25 mM. Germination capacity was reduced by Pb ions for the 12.5 mM concentration, germination was slightly reduced by both Pb compounds, while in concentrations of 25 mM of Pb(NO_3_)_2_ the germination rate was less than 40% of the control; however, with a 25 mM concentration of PbCl_2_, the germination rate was around 50% (Table 1A and 1B).

**Table 1:** Different parameter measured during expose of Scots pine seed to Pb compound [A- lead nitrate Pb(NO_3_)_2_ and B- lead chloride PbCl_2_], germination showed in percent, root length in centimeter, electrolyte leakage, superoxide anions (O_2_^•−^) in OD A_530_ x g^-1^ FW, g Glutathione half-cell reduction potential (E_GSSG/2GSH_) and mV—value show as mV.

**A**							
**Pb(NO**_**3**_**)**_**2**_	**Day**	**Germination**	**Root length**	**Electrolyte leakage**	**O**_**2**_^**-**^	**GSH/GSSH**	**mV**
Control	1	0 e	0 d	122.27 ±6.64 h	0.26 ±0.05 e	4.95 ±0.17	-386.14 ±0.62
	3	7 ±0.67 d	0.03 ±0.01 c	213.57 ±0.51g	0.38 ±0.01 d	2.78 ±1.59	-359.41 ±26.05
	7	74 ±1.33 a	1.63 ±0.08 a	231.70 ±2.07 d	0.46 ±0.01 c	1.74 ±0.15	-358.41 ±2.12
12.5 mM	1	0 e	0 d	213.73 ±1.38 g	3.80 ±0.02 e	4.23 ±0.02	-383.08 ±0.35
	3	1.33 ±1.78 e	0 d	281.20 ±5.20 e	3.82 ±0.07 d	1.90 ±0.17	-368.03 ±1.47
	7	67.33±4.89 b	0.28 ±0.01 b	447.17 ±4.49 c	5.83 ±0.11 d	0.55 ±0.01	-342.28 ±0.46
25 mM	1	0 e	0 d	321.90 ±4.27 f	4.20 ±0.03 e	3.70 ±0.11	-379.46 ±1.32
	3	0 e	0 d	377.93±8.29 b	3.93±0.09 b	1.60±0.10	-365.44±1.65
	7	42.67 ±2.44 c	0.17 ±0.01 b	524.03 ±7.71 a	6.15 ±0.19 a	0.56 ±0	-342.58 ±0.10
**B**							
**PbCl**_**2**_	**Day**	**Germination**	**Root length**	**Electrolyte leakage**	**O**_**2**_^**-**^	**GSH/GSSH**	**mV**
Control	1	0 e	0 e	122.27 ±6.64 e	0.26 ±0.05 d	4.95 ±0.17	-386.14 ±0.62
	3	7 ±0.67 d	0.04 ±0.01 d	213.57 ±0.51 cd	0.38 ±0.01 cd	2.78 ±1.59	-359.41 ±26.05
	7	74 ±1.33 a	1.63 ±0.08 a	231.70 ±2.07 c	0.46 ±0.01 ab	1.74 ±0.15	-358.41 ±2.12
12.5 mM	1	0 e	0 e	227.73 ±0.49 d	0.28 ±0.05 b	3.20 ±0.04	-376.99 ±0.46
	3	0.67 ±0.89 e	0 e	301.03 ±18.02 b	0.29 ±0.02 cd	1.91 ±0.28	-359.98 ±2.68
	7	70 ±2.67 b	0.27 ±0.01 b	316.09 ±4.61 a	0.35 ±0.04 b	0.34 ±0.01	-330.06 ±0.82
25 mM	1	0 e	0 e	244.40 ±5.33 cd	0.42 ±0.04 a	2.17 ±0.06	-369.11 ±0.63
	3	e	0 e	362.27 ±5.31 b	0.38 ±0.01 bc	1.12 ±0.06	-359.98 ±1.04
	7	53.67 ±1.11 c	0.17 ±0.01 c	359.73 ±11.18 a	0.41 ±0.02 ab	0.33 ±0.03	-331.35 ±2.21

Value measured for *P*. *sylvestris* seeds under control, or Pb treatment in a concentration of 12.5 mM, 25 mM after 1, 3, and 7 days of germination test, ANOVA, Duncan Test p≤0.05.

The concentrations of Pb compounds change the dynamic of root growth (Table 1). With a Pb concentration on 12.5 mM, the root length was 7 times shorter than in the control. The addition of Pb(NO_3_)_2_ inhibited root growth, while increasing concentrations of PbCl_2_ led to a reduction in root growth.

### Membrane damage

The addition of Pb ions was negatively correlated with electrolyte leakage. Besides the germination rate, the control and 12.5 mM Pb variant differ by a 70–60% increase in leakage of electrolytes (Table 1). Pb(NO_3_)_2_ causes more intense electrolyte leakage than PbCl_2_.

**Fig 1:**
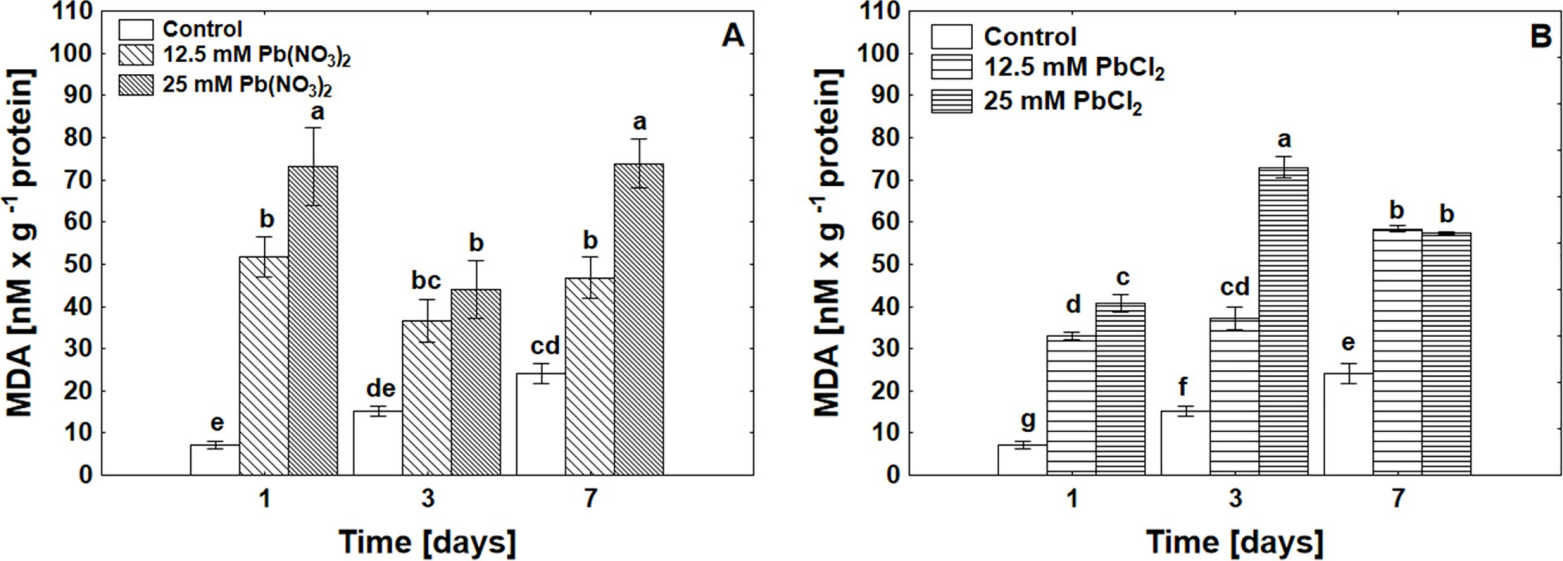
Malondialdehyde (MDA) content in *P*. *sylvestris* seeds under control, Pb(NO_3_)_2_ (A) or PbCl_2_ (B) treatments in concentrations of 12.5 mM and 25 mM after 1, 3 and 7 days of germination, ANOVA, Duncan Test p≤0.05.

An increasing concentration of Pb was correlated with an enhanced MDA level (Fig 1). A rise in the MDA level, i.e., increased permeability of cell membranes, was significantly visible at a concentration of 25 mM PbCl_2_ and Pb(NO_3_)_2._ No significant differences in MDA levels were observed when different salts were used.

### Oxidative stress parameters

#### Reactive oxygen species

**Fig 2:**
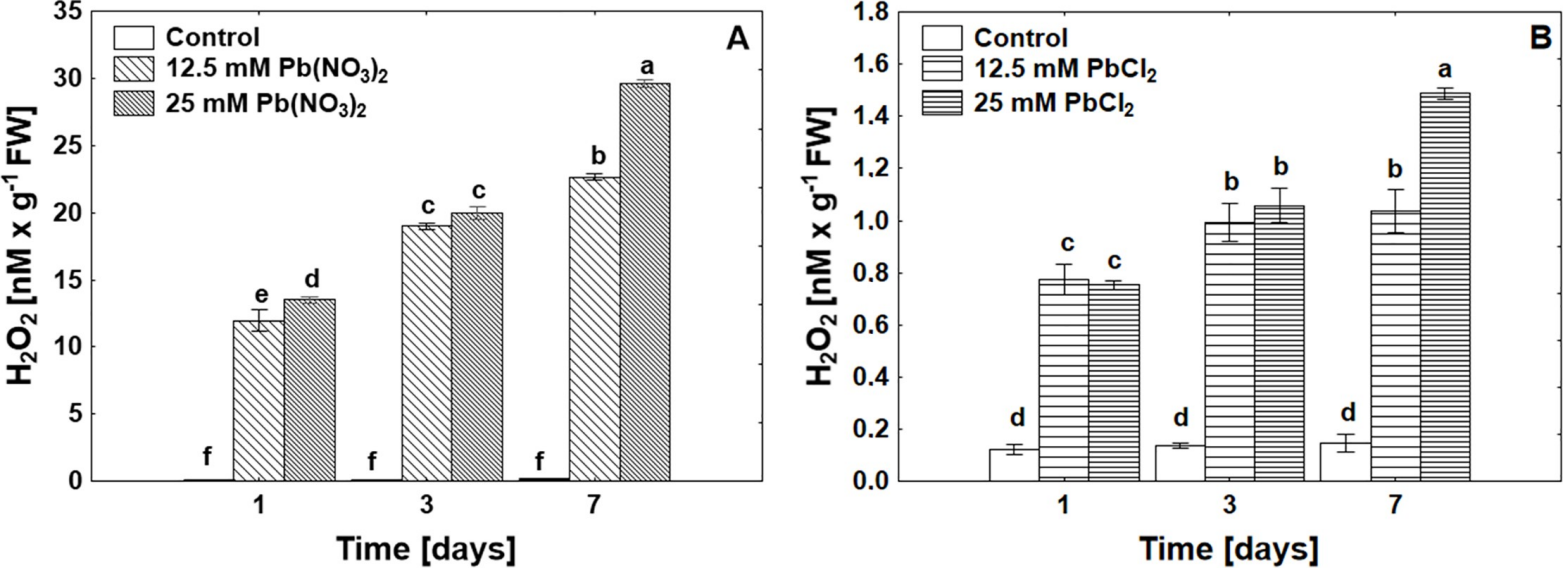
Hydrogen peroxide (H_2_O_2_) levels in *P*. *sylvestris* seeds under control, Pb(NO_3_)_2_ (A) or PbCl_2_ (B) treatments in concentrations of 12.5 mM and 25 mM after 1, 3 and 7 days of germination, ANOVA, Duncan Test p≤0.05.

Changes in oxidative levels in seeds under the influence of Pb are reflected in the rising level of H_2_O_2_ and O_2_^•−^. The level of O_2_^•−^radical content was positively correlated in each concentration of Pb(NO_3_)_2_. The amount of O_2_^•−^radical was eight times higher in lead nitrate treatments than in each concentration of PbCl_2_ in the experiments (Table 1). Pb(NO_3_)_2_ shows more toxic effects than PbCl_2_ and more H_2_O_2_ was produced during germination (Fig 2). The level of H_2_O_2_ in the control treatment was on low and constant level according to Pb treatment.

Visualisations of O_2_^●-^ and H_2_O_2_ showed that _i_n the control, treatment staining of all root segments was observed, while in the experimental variant, we observed intense staining of the root meristematic section (Supplementary S1 and S2 Figs).

### Catalase activity

**Fig 3:**
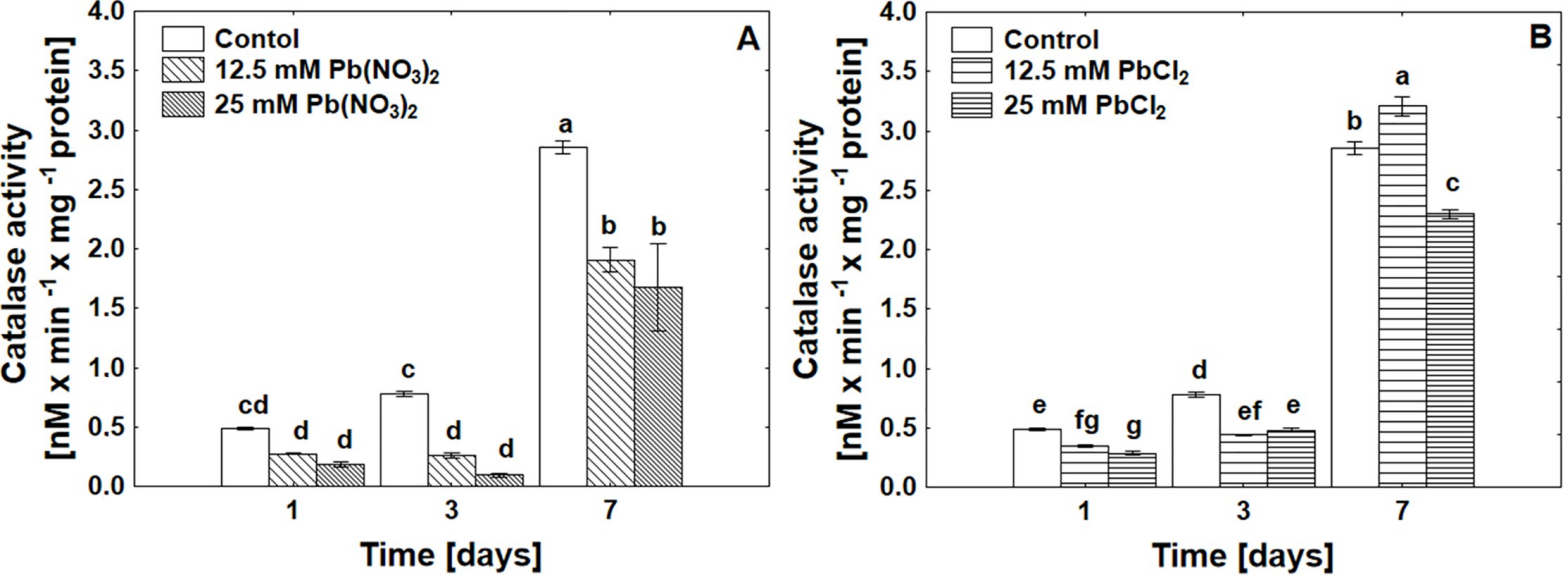
Catalase activity in *P*. *sylvestris* seeds under control, Pb(NO_3_)_2_ (A) or PbCl_2_ (B) treatments in concentrations of 12.5 mM and 25 mM after 1, 3 and 7 days of germination, ANOVA, Duncan Test p≤0.05.

Despite high levels of oxidative stress parameters, the activity of CAT (Fig 3) increases tremendously after 7 days of treatment as a result of *de novo* synthesis. This suggests that the activity of CAT is related to germination rates (Table 1) and the detoxification of H_2_O_2_ at the end of germination processes.

### Redox state

Glutathione regulates the redox balance. The high level of GSH in seeds treated with low Pb concentration may correspond with a new synthesis of GSH. The E_GSSG/2GSH_ increased in the third and seventh days, from -168mV, indicating changes in the redox state of the seeds’ cells (Table 1). The glutathione half-cell reduction potential (E_GSSG/2GSH_) was calculated based on the concentration of the reduced and oxidative forms of glutathione. At the beginning of the analysis, E_GSSG/2GSH_ was proximally from -195mV to -180mV, and lower in seeds treated with Pb(NO_3_)_2._ The GSH level decreased in seeds, and an increase in GSSG levels was observed (Table 2).

**Table 2:** Changes in the contents of reduced (GSH)–(A) and oxidised (GSSG)–(B) forms of glutathione of *P*. *sylvestris* seeds under lead nitrate PbCl_2_ and lead nitrate Pb(NO_3_)_2_ treatments in a concentration of 12.5 mM, 25 mM after 1, 3, and 7 days of germination, ANOVA, Test p≤0.05.

	A-GSH				B-GSSG		
Day	1	3	7		1	3	7
**Control**							
12.5 mM Pb(NO_3_)_2_							
25 mM Pb(NO_3_)_2_							
**Control**							
12.5 mM PbCl_2_							
25 mM PbCl_2_							
							
							
**Value**	minimal			control		maximal	

## Discussion

Pb is one of the heavy metals that significantly affects the metabolism of plant organisms [33]. The literature contains reports about the negative effects of metals on whole plants, but we wanted to study the defence mechanisms of the most important generative organs of plants, i.e. seeds. The main objectives of our research were to examine the effect of Pb on germination and the emergence of pine seedlings and to determine the degree of phytotoxicity of various Pb compounds: Pb(NO_3_)_2_ and PbCl_2_. Different chemical forms of Pb affect plants, change their biological properties, and are taken from the ground, transposed and accumulated in plant organs, ultimately leading to death [34, 35]. The authors [34] stated that Pb bioavailability/phytotoxicity also grew with increasing Pb forms and concentrations differently applied. The Pb toxicity due to various inorganic Pb salts was found in the decreasing order of Pb(NO_3_)_2_> PbCl_2_> PbSO_4_. The toxicity of Pb for plants was even higher with Pb at 100 μM as Pb(NO_3_)_2_ than the same concentration of applied Pb as PbCl_2_ and PbSO_4_, separately. The concentration of heavy metals in solution depends on the nature of inorganic ligand ions (e.g., HPO_4_^2-^, NO_3_^-^, Cl^-^ and SO_4_^2-^) and the pH of the solution, which ultimately govern the heavy metal sorption processes [36]. The presence of complexing ligands can increase metal retention or greatly increase metal mobility. The inhibitory effect of Pb ion on seed germination would be an undesirable factor, especially for tree species, e.g. Spruce (*Pice aabies* L. H. Karst.) or Scots pine, which have important applications in the forestry industry.

In our experiment, *P*. *sylvestris* seeds were treated with Pb in the form of salt: Pb(NO_3_)_2_ and PbCl_2_ in two concentrations of 12.5 mM and 25 mM each. Based on the obtained data, we found that Pb in both forms inhibited seed germination, whereas germination energy was lower when Pb(NO_3_)_2_ was used (Table 1). Earlier studies also suggest a reduction in seed germination under the influence of heavy metals on the selected permeability of cell membrane behaviour and osmotic changes [37], which is associated with reduced activity of digestive enzymes, such as amylase and protease, [38]. The authors [37] showed that Pb inhibited tomato germination by 45% using 220 ppm lead, with a slight Pb effect on the meristematic cells of the studied crops in the area of cell division, in contrast to metals such as Cu and Cd. Earlier retardation of radical emergence by Pb was also noted in *Brassica capestris* seeds [39]. An important phytotoxicity parameter is the root elongation test, which shows whether the growth process can be continued and whether the next stage of seedling emergence is possible. Literature data indicate that pine seeds were much more sensitive to Pb toxicity than Norway spruce, *Thespesia populneoides* (Roxb.), *Leucaena leucocephala* (Lam.) De Wit and *Delonix regia* Bojer Raf., [13, 14]. We observed a change in root growth dynamics under the influence of Pb in both chemical forms (Table 1). The root elongation was most inhibited by the Pb(NO_3_)_2_ salt over seven fold compared to control plants. Heavy metals might cause an inhibition of root growth that alters water balance and nutrient absorption and decreases calcium uptake in root tips, leading to a reduction in cell division or cell elongation [40]. In our research, we observed that in Scotch pine, germination inhibition occurs after 3 days of exposure to various concentrations of Pb chemical compounds, through changes in oxidative stress levels were noticed from the beginning of germination processes. The presence of Pb ions was negatively correlated with electrolyte leakage. In addition, the electrolyte leakage was three times higher for Pb(NO_3_)_2_ than for PbCl_2_ treatment (Table 1). As a result of exposure to Pb ions, we observed an increase in superoxide anion radicals and hydrogen peroxide levels in *P*. *sylvestris* seed cells, the highest levels were generated by Pb(NO_3_)_2_ especially after prolonged exposure to metal (Table 1). Generation of O_2_^.-^ and H_2_O_2_ in pine seeds and cuttings was confirmed by histochemical visualizations (S1 and S2 Figs). Other authors also showed using a histochemical examination an increase in O_2_^.-^ and H_2_O_2_ in poplar roots and leaves treated with lower Pb concentration (50 μM PbCl_2_) [41]. A tremendous increase in the level of H_2_O_2_ was observed in rice reaching up to 150% during the 400, 800 and 1200 ppm of Pb(NO_3_)_2_ treatment [42]. Along with the increase in the level of ROS in seed cells, we observed a higher level of MDA in seeds treated with two Pb compounds than in the control, although no significant differences were found when different Pb salts were used (Fig 1). MDA level showed a negative correlation, the level increased due to time of exposure (Fig 1) with increased electrolyte leakage (Table 1), This suggest that Pb exposure in each treatment led to membrane damage and reflects the ROS level. In *Festuca arundinacea* Schreb. seed under Pb and CD treatment, the MDA level varied during time of exposure (at the beginning and late time of germination showed a high amount compared to the lower level in the middle of the germination test) [43]. Changes in the level of oxidation reflect the state of the seeds, which is indicated in the viability of the seeds, however nevertheless the seed coating can be the first stage of the system of defence against toxic ions [8].

We determined the activity of catalase, one of the main antioxidant enzymes with the ability to convert H_2_O_2_ to H_2_O and O_2_ in an energy efficient way unlike other antioxidant enzymes, which use reducing equivalents [37]. In seed cells we found, especially after seven days of exposure to lead, a tremendous increase in CAT activity (Fig 3), likely as a result of *de novo* synthesis of enzyme protein. We noticed that Pb(NO_3_)_2_ used at the same concentration as PbCl_2_ is definitely more phytotoxic. This is manifested not only by the generation of higher levels of ROS, but also by the greater inhibition of CAT activity. It turns out that Pb(NO_3_)_2_ at concentrations of 12 mM and 25 mM causes catalase inhibition at the level of activity or synthesis.

**Fig 4:**
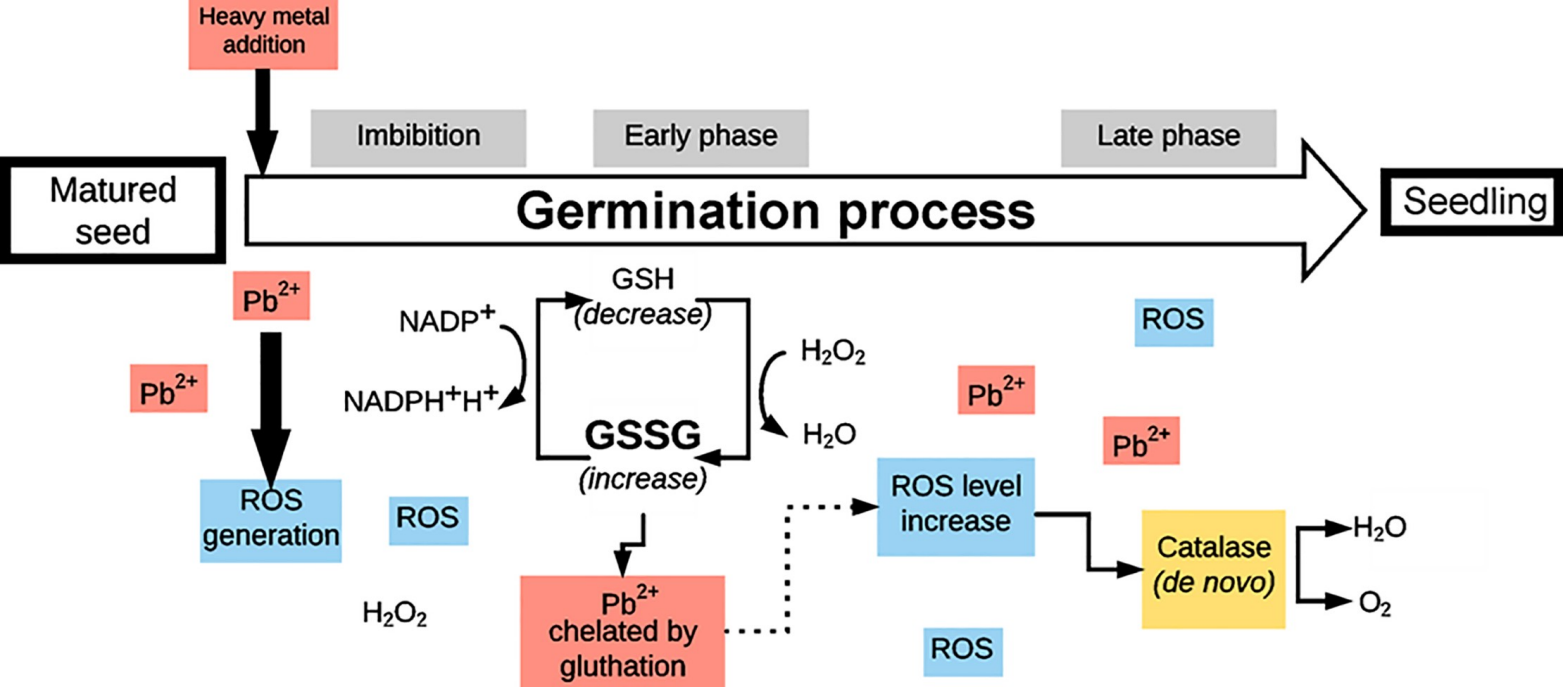
Pb ions in *P*. *sylvestris* L. seed cause a reaction in the antioxidant system. Ions added at the stage of imbibition in the first stage activate the non-enzymatic antioxidant response (via GSH) but when the germination processes are at an advanced stage, enzymatic (via CAT) antioxidants play a key role.

We observed that, the increase in CAT activity in the remaining variants is associated with germination rates (Table 1) and, as other authors indicate, H_2_O_2_ detoxification at the end of germination processes [28, 44]. Some authors [37] demonstrated high CAT activity in peas treated with 220 ppm Pb(NO_3_)_2_. Also an increase in CAT activity was observed after 1 day of PbCO_3_ treatment in *P*. *massoniana* in needles, stems and roots; was 4.3, 8.9 and 7.8 times higher, respectively, than in the control group [45]. In plant seeds, the defence mechanism consists of a detoxification and antioxidant system. Glutathione is involved in antioxidative defence by regulating the redox state, and has a protective function in the detoxification of heavy metals [46]. GSH is a precursor to phytochelatins synthesised in response to the presence of heavy metals that bind complexes are then transported to vacuoles [47]. In our research, we confirmed the participation of glutathione especially at the beginning of germination processes (Table 1). When using a lower concentration of PbCl_2_, we observed an increase in the level of the reduced form of glutathione GSH, associated with *de novo* synthesis [48]. With the increase in Pb exposure time, in the form of both nitrate and chloride, we observed an increase in the level of the GSSG oxidized form indicating the presence of oxidative stress conditions and changes in redox balance in seeds (Fig 4) [19, 20].

The use of Pb(NO_3_)_2_ in *P*. *massoniana* seeds increased the level of GSSG in stems and roots by 15% and 12%, respectively, compared to the control after 1 day of application [45]. The reduced GSH and GSH/GSSG ratio might be attributable to a higher consumption of GSH not only as an antioxidant but also for the synthesis of phytochelatins which reduced the free Pb content in the cytoplasm [45]. Other authors [49] also observed that the GSH concentrations were ~ 1–5.7 times higher in the roots of PbCl_2_ treated poplars in comparison to those not treated with Pb under low or high sulphur conditions. With the transition of lead exposure to seeds, the redox potential increased and shifted towards pro-oxidative reactions, indicating redox changes in seminal cells (Table 2). Such negative potentials are typical for metabolically active cells [20]. To summarise, our studies showed that the use of Pb in both the nitrate and chloride forms induced oxidative stress in *P*. *sylvestris* L. seeds, which triggered defence mechanisms. Pb(NO_3_)_2_ turned out to be a much more phytotoxic form compared to PbCl_2_, although both forms are equally labile fractions. Perhaps the Pb in the form of nitrate is more bioavailable for plants. Nitrate is the major source of nitrogen for most terrestrial plants, which serves as both an essential nutrient and a signal molecule involved in plant metabolism, growth, development, and adaptation to various environments [50]. Due to the presence of different concentrations of nitrate in the soil, plants have developed several membrane proteins that are found to be involved in the absorption of nitrate from the external environment into plants, and its transportation and translocation between different parts of the whole plant. In addition to nitrate, they can transport a diverse range of substrates, enabling their participation in diverse biological processes including plant growth, development, and adaptation to complicated environments [50]. Perhaps due to the numerous protein membrane transporters, Pb(NO_3_)_2_ is easier for plants to load and transport and thus induces a greater defence response. However, there are authors [51] who reported that the effect of Pb and other metals (Cu and Zn) on some cereal crops (ie, barley, wheat and rice) seedlings was more pronounced and was not due to the presence of: SO_4_^2-^ or Cl^-^ ions. The data collected so far indicate the need for further research aimed at determining the phytotoxic effects of heavy metals on plants.

## Conclusions

Viability of Scots pine seed germinated under heavy metal was found to remain at a high level due to the sequencing of non-enzymatic (GSH) and enzymatic (CAT) cycles of ROS detoxification.

The level of GSH during the early stages of germination increased in each lead-treatment condition. Under late germination stages the reduced form of GSH decreased but GSSG levels increased.

In pine seeds CAT is probably synthetized *de novo* and in the late phase of germination CAT play an important role in ROS scavenging and seedlings development.

Pb(NO_3_)_2_ turned out to be a much more phytotoxic form compared to PbCl_2_, although both forms are equally labile fractions. The form of Pb nitrate is more bioavailable for plants.

## Supporting information

S1 Fig Superoxide (O_2_^.-^) _-_localisation (with nitrobluetetrazolium chloride) in *P*. *sylvestris* seeds under control (A), PbCl_2_ (B– 12.5 mM) or Pb(NO_3_)_2_ (C– 12.5 mM) after 1,3,7 days of treatment.
(TIF)

S2 Fig Hydrogen peroxide (H_2_O_2_) localisation (with 3,3’-diaminobenzidine) levels in *P*. *sylvestris* seeds under control (A), PbCl_2_ (B– 12.5 mM) or Pb(NO_3_)_2_ (C– 12.5 mM) after 1,3,7 days of treatment.
(TIF)
