# Supplementary figures and images for: Differences in stress defence mechanisms in germinating seeds of Pinus sylvestris exposed to various lead chemical forms
Source: PLoS One. 2020 Sep 28;15(9):e0238448. doi: 10.1371/journal.pone.0238448 (PMC7521717; doi:10.1371/journal.pone.0238448)

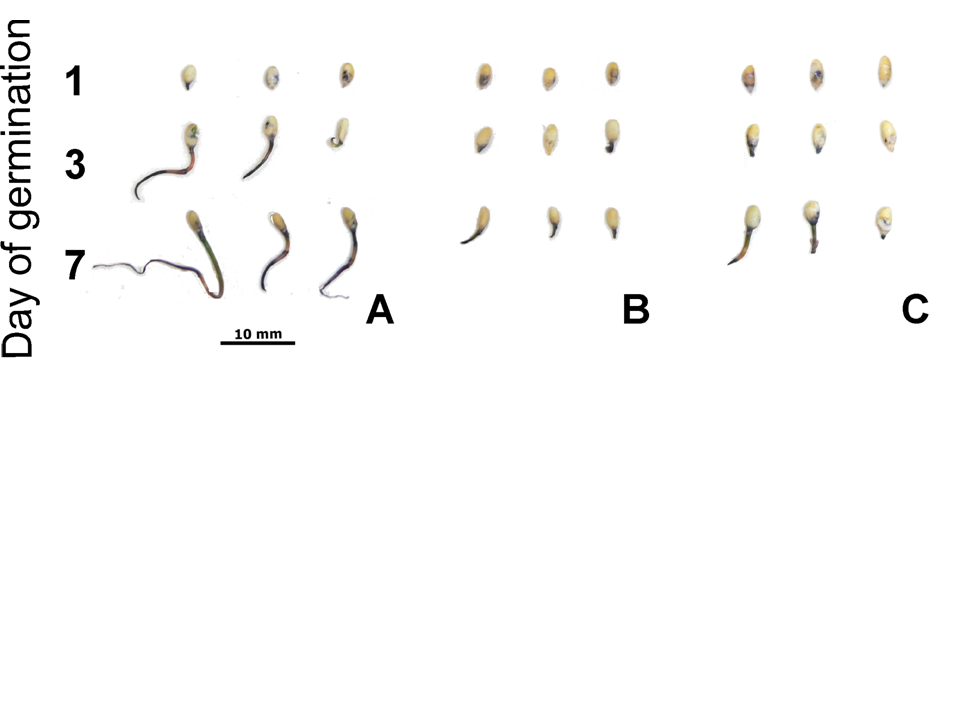

Supplement: S1 Fig — Superoxide (O2.-) -localisation (with nitrobluetetrazolium chloride) in P. sylvestris seeds under control (A), PbCl2 (B– 12.5 mM) or Pb(NO3)2 (C– 12.5 mM) after 1,3,7 days of treatment. (TIF) [file pone.0238448.s001.tif]

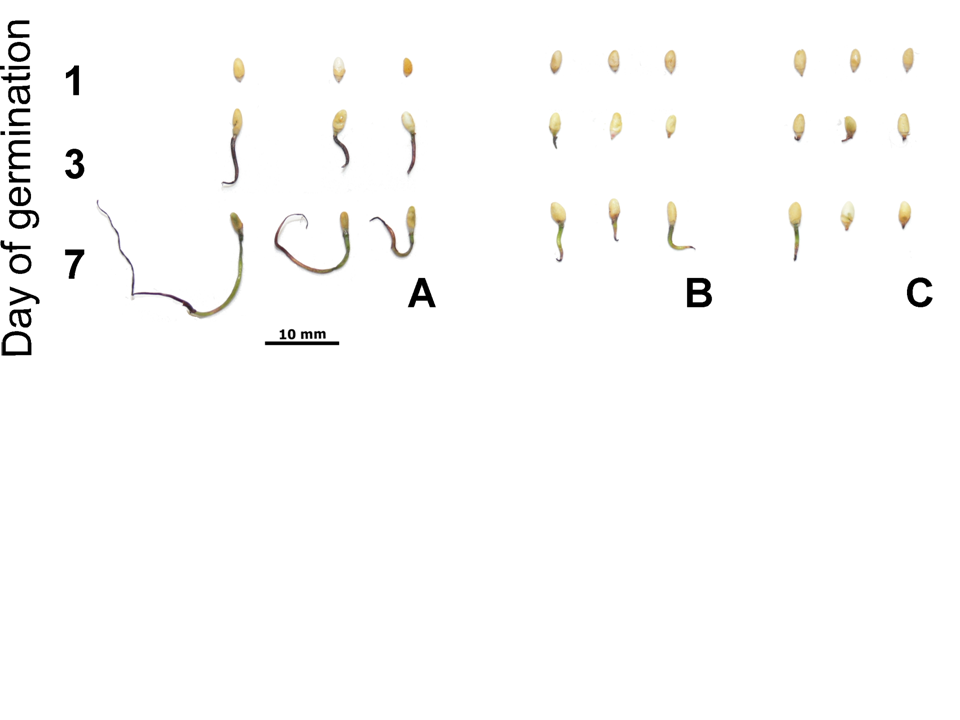

Supplement: S2 Fig — Hydrogen peroxide (H2O2) localisation (with 3,3’-diaminobenzidine) levels in P. sylvestris seeds under control (A), PbCl2 (B– 12.5 mM) or Pb(NO3)2 (C– 12.5 mM) after 1,3,7 days of treatment. (TIF) [file pone.0238448.s002.tif]
